# Hydrogels: 3D Drug Delivery Systems for Nanoparticles and Extracellular Vesicles

**DOI:** 10.3390/biomedicines9111694

**Published:** 2021-11-15

**Authors:** Yashna Chabria, Garry P. Duffy, Aoife J Lowery, Róisín M. Dwyer

**Affiliations:** 1 Discipline of Surgery, Lambe Institute for Translational Research, National University of Ireland Galway, H91 V4AY Galway, Ireland; y.chabria1@nuigalway.ie (Y.C.); aoife.lowery@nuigalway.ie (A.J.L.); 2 CÚRAM, The SFI Research Centre for Medical Devices, National University of Ireland Galway, H91 W2TY Galway, Ireland; garry.duffy@nuigalway.ie; 3 Anatomy & Regenerative Medicine Institute, School of Medicine, College of Medicine Nursing and Health Sciences, National University of Ireland Galway, H91 TK33 Galway, Ireland

**Keywords:** nanoparticles, extracellular vesicles, hydrogels, biocompatible, bioscaffolds, tissue regeneration, cancer

## Abstract

Synthetic and naturally occurring nano-sized particles present versatile vehicles for the delivery of therapy in a range of clinical settings. Their small size and modifiable physicochemical properties support refinement of targeting capabilities, immune response, and therapeutic cargo, but rapid clearance from the body and limited efficacy remain a major challenge. This highlights the need for a local sustained delivery system for nanoparticles (NPs) and extracellular vesicles (EVs) at the target site that will ensure prolonged exposure, maximum efficacy and dose, and minimal toxicity. Biocompatible hydrogels loaded with therapeutic NPs/EVs hold immense promise as cell-free sustained and targeted delivery systems in a range of disease settings. These bioscaffolds ensure retention of the nano-sized particles at the target site and can also act as controlled release systems for therapeutics over a prolonged period of time. The encapsulation of stimuli sensitive components into hydrogels supports the release of the content on-demand. In this review, we highlight the prospect of the sustained and prolonged delivery of these nano-sized therapeutic entities from hydrogels for broad applications spanning tissue regeneration and cancer treatment. Further understanding of the parameters controlling the release rate of these particles and efficient transfer of cargo to target cells will be fundamental to success.

## 1. Introduction

Nanoparticles (NPs) are nanoscale entities consisting primarily of lipid and polymeric structures. The ability of NPs to encapsulate components including proteins, peptides, nucleic acids and small molecules has resulted in widespread interest in their potential as targeted delivery agents for therapeutic drugs [1]. Over the last few years, the significance of nano-sized particles has escalated due to their potential use in the clinical setting for the treatment of various diseases as they can elicit site specific effects as well as be tuned for time-controlled release [2,3,4]. NPs can also be modified or engineered to elicit an enhanced therapeutic effect at the target site due to improved pharmacokinetics and pharmacodynamics, and active intracellular delivery [5]. Although NPs are known to be readily phagocytized by macrophages as an immediate host immune response to a foreign body, these limitations are now being overcome through the modulation of physicochemical properties such as surface charge, size, steric effects, and surface modifications.

For example, many nanoparticles are now being coated with different types of polymers in order to bypass the immune system as they form a hydrophilic coating [5,6]. Nanotherapeutics have the potential to ensure enhanced bioavailability, prolonged effects of the therapeutic gene or drug at the target site, as well as abated chemical/enzymatic degradation resulting in enhanced stability [7,8]. The commonly employed nanoparticles include biodegradable NPs, lipid-based NPs, polymeric NPs, and micelles that were found to be potential drug delivery systems. Among these, phospholipid-based vesicles known as liposomes have been prominent nanocarriers for the delivery of therapeutic cargo. This is due to the phospholipid bilayers that contain an internal hydrophilic compartment, supporting the ability to encapsulate hydrophilic as well as hydrophobic constituents effectively based on their synthesis [9,10]. The first nanomedicines to be approved for clinical use by the FDA were liposomes. Liposome formulations carrying anti-cancer drugs, such as doxorubicin and amphotericin B, were approved in the 1990s. Liposomes usually have shorter half-lives due to rapid clearance of these particles by circulating macrophages. PEGylation of NPs was found to minimize clearance and was adopted for the treatment of various malignancies, fungal infections, and macular degeneration [11,12,13,14]. A liposomal Irinotecan is the most recently approved drug carrier that works by passively targeting pancreatic cancer as a topoisomerase I inhibitor [11]. Over the years more than 25 inorganic NPs have also been approved for clinical use by the FDA, with the first one being Cosmo Fer for treating iron deficiency in 1974 [15].

Similar to the synthetic NPs, nature has its own nanoparticles known as extracellular vesicles (EVs), which are lipid bound vesicles secreted by almost all cell types into the extracellular space. Recent findings have highlighted the role of EVs in intercellular communication resulting in the transfer of a wide range of nucleic acids, peptides and proteins that are now being developed for the treatment of multiple diseases [16]. The composition of these vesicles is mainly governed by the cell of origin, the local niche, the pathways involved in biogenesis and the cargo sorting routes. “EVs” is an umbrella term that has been advocated for nano-sized vesicles including microvesicles, exosomes and apoptotic bodies, which are defined by their respective size and biogenesis pathway [17,18]. These vesicles resemble synthetic liposomes as they are bound by lipid membranes that provide protection against proteases and nucleases [19,20]. EVs are therefore an intense topic of research at the moment as they have emerged as potent carriers for the delivery of therapeutic cargo mainly because they are believed to be the fingerprint of the secreting cell, and ideally could bypass the immune system if isolated from immune-compatible cell sources [21]. The use of EVs also bears the potential to replace cell therapy and its associated complications such as undesirable immunological reactions, the transfer of mutated or damaged genetic material and transformation potential [20]. The small size of EVs enables easy transit across biological barriers, the potential to bypass the immune system and to home to the target site based on its surface modifications [20].

Currently, the majority (>30) of EV-based clinical trials focus on employing them as a tool for diagnosis of various diseases by isolating EVs from body fluids and detection of EV cargo indicative of disease. Based on recent reports there are >20 ongoing clinical trials that employ EV therapeutics for different diseases [22,23]. A recently completed randomized controlled study highlighted the efficacy of platelet rich plasma (PRP) containing EVs for the treatment of temporal bone cavities [24]. Another ongoing clinical trial is also focused on the use of PRP rich in EVs for the treatment of ear infections [25]. As there are many ongoing clinical trials with Mesenchymal Stromal Cells (MSCs) mainly attributing to their regenerative capacity, MSC-EVs have also been studied to analyze their potential for therapeutic benefit. A trial for the treatment of advanced stages of colorectal cancer involved subcutaneous administration of 2 × 10^12^ ascites-derived EVs weekly for a month and was found to be safe and effective by eliciting antigen-specific T lymphocyte response [26]. Another ongoing Phase I clinical trial for the treatment of metastatic pancreatic ductal carcinoma caused by a mutation in KrasG12D, involves treatment with MSC-EVs carrying siRNA to this KrasG12D mutation. Patients will be systemically injected with three doses of EVs biweekly for three courses and will be monitored for a year [27]. Several clinical trials employing MSC-EV treatment are now focused on the treatment of the coronavirus (SARS-COVID-2). Aerosol administration of 2 × 10^8^ vesicles/3 mL for a week is also being carried out on COVID-19 patients in China [28]. Although clinical trials results have not yet been released, multiple studies are ongoing, such as the phase-2 clinical trial for moderate to severe COVID-19, thereby highlighting the immense and broad potential of therapeutic EVs [29].

## 2. Limitations in the Current Route of Administration of NPs and EVs

Although NPs and EVs offer exciting potential as drug carrier systems, they come with certain limitations. When introduced in the body, they are subjected to several physical and biological obstacles that alter the dosage of the NPs/EVs reaching the target site. These blocks mainly include phagocytic sequestration, aggregation, harsh flow and shear forces, varying pH, diffusion, renal clearance, and much more [30]. The nano size and suitable structure on the one hand enables these particles to traverse multiple physiological barriers, but on the other hand results in rapid clearance from the body. Studies by Takahashi et al., revealed that B16-BL6 murine melanoma cell EVs are rapidly cleared from the circulation when administered via direct intravenous, intraperitoneal or subcutaneous injections and are found to be accumulated at non-specific sites such as the liver, spleen, gastrointestinal tracts, kidneys and lungs [31,32]. Wiklander et al. [33] also reported that the majority of injected exosomes were rapidly cleared by macrophages in the reticuloendothelial system. The clearance of exosomes by the immune system was found to vary considerably based on the route of administration and the different cell sources. Another study using exosomes for the treatment of pancreatic cancer indicated the ability of CD47 positive exosomes to evade phagocytosis by circulating monocytes, increasing their half-life in circulation [34]. Along with rapid clearance, another major limitation is the struggle of mass production of pure and consistent EVs [20,35]. Dynamic distribution also results in decreased retention of the drug at the target site as well as increased risk of exposing healthy tissues to undesired toxicity. These impediments associated with the systemic delivery of NPs/EVs limit the wide use of nanomedicines in the clinic [36]. This highlights the need for a local sustained delivery system at the target site that will ensure prolonged exposure, maximum efficacy and minimal toxicity [36]. In order to enhance the therapeutic index, many studies now focus on the establishment of a controlled drug delivery system that incorporates NPs/EVs into biomaterials [35,36,37,38,39,40,41,42].

## 3. Hydrogels as Reservoirs for Sustained Delivery of NPs/EVs

This review highlights the use of sustained delivery systems for the targeted delivery of NPs/EVs using highly porous biomaterials known as hydrogels. These have been extensively used as efficient drug delivery systems that ensure sustained release of the therapeutics encapsulated within. These 3D constructs of insoluble matrices are formed by the crosslinking of hydrophilic copolymers, macromers or homopolymers [43]. Hydrogel efficiency is attributed to the cross-linked networks of water-soluble polymers that provide a 3D structure that is highly porous, resulting in enhanced encapsulation of therapeutic particles as well as ensuring sustained release [43].

These hydrophilic polymers bear the potential to absorb water from minimal amounts up to a thousand times their dry weight giving them the ability to either be chemically stable or subsequently disintegrate. The commonly employed polymers for biomedical applications include synthetic hydrogels such as PEG (poly-ethylene glycol), PVA (Poly-vinyl alcohol) and PHEMA (poly(2-hydroxyethyl methacrylate)) or natural polymers such as collagen, hyaluronic acid, chitosan, alginate and agarose [44]. Most of the hydrogels commonly employed are biocompatible as they are produced from naturally occurring components of the human body such as collagen, hyaluronic acid, fibrin, dextran, and so forth [45]. Many studies have focused on the use of hydrogels for encapsulation of cells [46,47,48,49], however due to the limitations associated with cell therapies many approaches are now based on sustained drug delivery systems containing therapeutic NPs.

These have been incorporated into hydrogels through a variety of approaches, including the mixing of NPs with monomers, incorporating them into the polymeric solution. NPs can also be added to pre-made gels by the process of breathing, where hydrogels are soaked in a solution of NPs to enable their uptake by swelling, or by in-situ conversion where NPs are formed inside the hydrogels by the use of a precursor [50]. Previous studies have achieved sustained release of NPs by regulating the ratio between the mean mesh size of the hydrogels and the diameter of the NPs [51]. The retention and release of therapeutic proteins are mainly regulated by the mesh size of the hydrogels [52]; however, as hydrogels are not homogenous physical enmeshments, the crosslinked network is not sufficient for encasing the NPs. Therefore, many studies are now focused on electrostatic interactions that can be established in polymers for tuning the release kinetics [51]. Therapeutic drugs/NPs are retained in the hydrogel matrix by hydrophobic or charge interactions, hydrogen bonding, stereocomplexation, covalent crosslinking or small molecule cross linking [43]. The stability of NPs in the hydrogel system is also an important consideration. This has been addressed through alteration of the amount of crosslinkers used, and also through the use of surfactants or microgels [53]. Recent studies have reported the capacity of the side chains of synthetic hydrogels to enhance stability when their chemical functionality is altered [54]. The monomers or semi-polymer chains of hydrogels have also been reported to be key regulators of maintaining the size and shape of silver NPs [55]. The highly porous nature enables enhanced drug loading capacity along with the subsequent release from hydrogel complexes based on the diffusion coefficient of the small molecule [43]. The encapsulation of stimuli sensitive components into these “intelligent hydrogels” enables systemic release of the content on-demand. A popular example of such well controlled systems is the glucose-sensitive hydrogels that modulate insulin levels in patients based on the glucose concentration at that moment in order to maintain stable blood sugar levels at all times [37,56].

Hydrogels can also be formulated into different shapes and thickness based on requirements. As a result, these are now being extensively used in clinical practice as well as experimental medicine. Currently, 30 injectable hydrogels have been approved by the FDA with applications mainly focusing on skin regeneration, osteoarthritis and spinal cord regeneration [57]. Many synthetic hydrogels such as polyacrylamide gels as well as natural gels such as hyaluronan and hydroxyethyl cellulose are being investigated for the treatment of osteoarthritis [58]. Hydrogels have also been extensively investigated for ocular therapeutics; the only candidate for age-related macular degeneration (AMD) in clinical trials is the Ocular Therapeutix’s OTX-TKI (tyrosine kinase inhibitor microcrystals in PEG hydrogel) [57]. Compared to other groups of synthetic biomaterials, hydrogels are very similar to the living tissues of the human body due to their water absorption capacity. Due to this they possess the unique ability to undergo various alterations in the gel structure such as swelling, dissolution or degradation that results in sustained release of the therapeutic particles upon external stimuli, making them responsive to factors in the microenvironment such as temperature and pH, thereby making them potential candidates for sustained drug delivery systems. Advances in hydrogel technologies have spiked over the past few decades for several biomedical applications especially those involving sustained drug delivery. Therefore, many novel hydrogel based delivery systems have been established with multiple small molecule drugs, NPs and EVs to fulfil the escalating demand of the pharmaceutical and medical industry (Table 1).

## 4. Sustained Delivery of NPs/EVs for Tissue Regeneration

**Figure 1:**
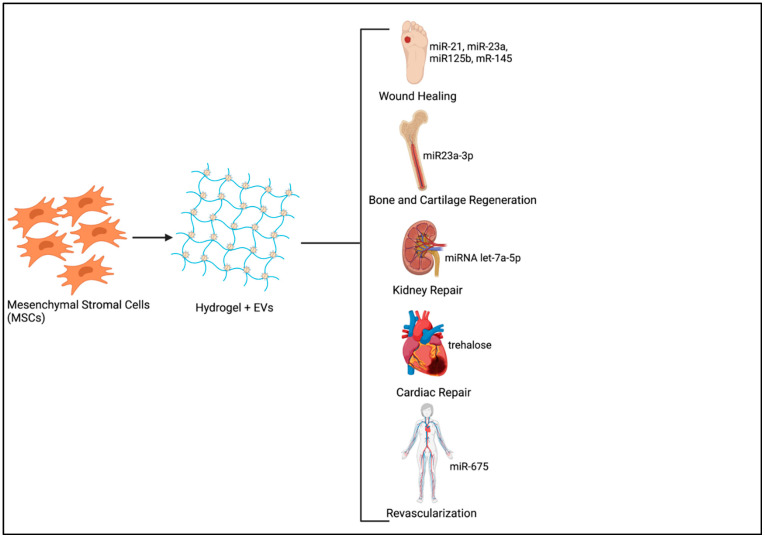
Sustained Delivery system of MSC derived Extracellular vesicles (EVs) for Tissue Regeneration. (Image created using www.Biorender.com-paid subscription, accessed on 29 June 2021, Biorender 2021).

Harnessing the body’s own regenerative capacity has been extensively researched over several decades. Many studies have focused on the use of different therapeutics for triggering endogenous regeneration, such as instructive biomaterial scaffolds, NPs, small molecules, and so forth [72]. Therapeutic NPs have been exploited for potential regenerative applications mainly due to their versatility in terms of altering surface chemistry, size and potential to be used as delivery vehicles for the transfer of drugs, growth factors, small molecules, or genetic material [73,74]. Tissue repair and regeneration require extended periods of exposure to therapeutic molecules with increased uptake of these substances at the injured site by endocytosis being crucial for exerting the expected biological effects. The encapsulation of EVs in a hydrogel introduced at the tissue defect is therefore the best way forward as it not only enhances EV half-life by minimizing non-specific binding at other sites, but also ensures optimal therapeutic effects at minimal doses [44,75] (Figure 1).

### 4.1. Wound Healing

Limited systemic treatments are available for chronic injuries such as diabetic wounds mainly due to dysregulation of the cellular response as well as high incidence of infections, therefore novel therapeutic approaches are constantly being developed. The delayed healing not only results in infections such as gangrene and eventual amputation of the infected organ but can also be lethal in severe cases. The conventional treatment with limited success includes antibacterial dressings with plasters and biocompatible hydrogels [40,76,77,78]. Multifold studies over the years have highlighted the regenerative capacity of metal NPs especially gold and silver NPs mainly attributing efficacy to the tunable size and surface characteristics.

For example, sustained release of silver NPs through polymeric hydrogels has been found to be promising for wound healing due to the antimicrobial and healing properties [64,74]. Hydrogels responsive to varying pH have also been found to be effective drug delivery systems for wound healing. A recent study highlighted the potential of silver NPs loaded in pH responsive hydrogels as a potential candidate for wound healing. This was due to the unique property of switching to a hydrated state in the alkaline wound environment, resulting in the sustained release of silver NPs with antibacterial properties [65]. Another study assessing the efficacy of gold nanorods released from different hydrogels namely polyethylene glycol (PEG) and cationic poly allyl amine hydrochloride (PAH) demonstrated prominent wound healing ability with complete closure of the wound within 14 days of the treatment along with improved skin re-epithelialization and collagen formation as well as noticeable impact on the inflammatory gene expression [79]. Combination hydrogel composed of chitosan and poly-vinyl alcohol dispensing heparinized zinc NPs has been considered as an effective wound dressing material attributing to its enhanced antibacterial and rapid wound healing properties with complete wound closure within 14 days [66]. Another study highlighted the immunomodulatory effects of MSC exosomes when delivered using polymeric fibrous scaffolds in a murine model. The scaffolding material and exosomes were found to attract immune cells by modulating macrophage, T helper cell and T regulatory cell responses and in turn promoting tissue repair [80].

Stable EV delivery systems comprising of biocompatible hydrogels are also being extensively studied for their therapeutic regenerative capacity. EVs derived from Human-Gingival MSCs (hGMSC-EVs) when incorporated into chitosan/silk hydrogel sponge were found to significantly promote wound healing by augmenting re-epithelialization along with extracellular matrix (ECM) deposition and remodeling, and angiogenesis in a diabetic rat model [40]. Another study using commercially available hydro-matrix hydrogel highlighted the wound healing ability of sustained exposure to Human Umbilical cord derived MSCs (hUCMSC-EVs) enriched with microRNAs (miR-21, miR-23a, miR-125b and miR-145). Prolonged exposure to these EVs resulted in a significant decline in scarring as well as myofibroblast formation in the in-vivo model [81]. Adipose stromal cell derived EVs (ADSC-EVs) when encased in alginate hydrogels were also found to elicit similar wound healing properties by not only improving the wound closure and promoting angiogenesis, but also enhancing collagen deposition at the injured site [82].

### 4.2. Bone and Cartilage Regeneration

The demand for tissue engineering solutions for effective bone and cartilage regeneration has escalated over the years due to an aging population with associated rapid decline in bone healing ability. Bio-printed 3D scaffolds have been observed to provide the required mechanical support that accelerates bone regeneration [83,84]. Biocompatible hydrogels used as reservoirs of therapeutic NPs and growth factors have also been assessed for their efficacy in bone regeneration. A significant increase in bone regeneration was also observed in a nanofiber scaffold of poly(ε-caprolactone) (PCL) and poly(glycerol sebacate) (PGS) packed with hydroxyapatite NPs (HANPs) and simvastatin (SIM) with a significant increase in mineralization and bone formation [85]. Thermosensitive chitosan based hydrogels were found to bear notable potential for continuous local delivery of stromal cell-derived factor-1α (SDF-1α) loaded NPs for the treatment of calvarial defects [67].

Studies have previously confirmed the clinical efficacy of chitosan hydrogels for the delivery of growth factors and soluble factors to promote chondrogenic regeneration [86]. Synthetic hydrogels have also been investigated as potential therapy for articular cartilage damage. To simulate the lubrication of cartilage, which is critical to function, Lin et al. [87] employed multilamellar vesicles (MLVs) to incorporate phosphatidylcholine (PC) dimyristoylphosphatidylcholine (DMPC) or hydrogenated soy (HSPC) lipids into synthetic hydrogels. The lipids were distributed through microreservoirs to support the continuous replenishment of lubrication as they wear. This lipid incorporation was found to result in a 95%–99% reduction in friction [87]. This promising data support the potential of self-lubricating hydrogels for reduction of the impact of friction and wear [87]. Improved cartilage repair was observed with a composite hydrogel system of silk fibroin loaded with BMP-2 as well as chitosan NPs containing TGF-β1. The in vitro and in-vivo models not only displayed enhanced biocompatibility but also improved chondrogenesis at the cartilage defect when compared to the hydrogel alone [68].

Stem cell derived EVs were found to improve osteogenic differentiation by enhancing osteogenic gene expression through paracrine signaling factors. MSC derived EVs from dental tissues have also been observed to promote skeletal regeneration when administered to the damaged bone site for prolonged time frames using different scaffolding biomaterials [41]. Studies using synthetic Poly-Lactide Scaffold (PLA) or natural collagen scaffold for assessing the prolonged effects of therapeutic EVs over a period of 6 weeks also demonstrated pro-osteogenic alterations at the site of damage. The sustained exposure to EVs not only supported formation of new bone nodules and increased mineralization by having an effect on the osteogenic genes, but also promoted the vascular network that resulted in rapid bone regeneration [41,88]. Complex combination hydrogels comprising of coralline hydroxyapatite, silk fibroin, glycol chitosan along with synthetic polyethylene glycol (PEG) for the delivery of hUCMSC-EVs were found to accelerate bone ossification by escalating BMP-2 and collagen deposition and promoting angiogenesis. The factors contributing to the sustained delivery of EVs were the uniform pore size and connectivity in the hydrogel along with its hydrophilicity, making it an attractive biomaterial as the therapeutic effects were observed over a period of 90 days at the site of injury [89].

With limited success through innate healing, the clinical demand for therapeutic options for articular cartilage repair is ever increasing. Articular cartilage lesions were significantly restored when treated with induced pluripotent stem cell cells (iPSC)-derived exosomes integrated with a Photoinduced Imine Crosslinking (PIC) hydrogel [35]. The exosome-hydrogel tissue patch not only maintained a consistent supply of EVs but also integrated with the native cartilage cells over a period of 14 days making this acellular hydrogel glue scaffold a promising candidate for cartilage regeneration [35]. MSC-EVs enriched with miR-23a-3p delivered using a Gelma/nanoclay hydrogel were also found to significantly promote cartilage regeneration by activating PTEN/AKT signaling pathway [90].

### 4.3. Vascularization and Cardiac Repair

Consistent supply of oxygen and nutrients is essential for the regeneration and maintenance of cells and tissues. Scaffolds promoting vascularization have thereby been extensively developed in order to provide the required support that would mimic the native extracellular matrix (ECM). However, these lack biological constituents that would enhance revascularization [42]. Employing synthetic NP delivery based hydrogel systems have shown significant effects in promoting revascularization by sustained local delivery of angiogenic factors such as VEGF. The delivery of free VEGF using a hydrogel resulted in rapid release over a period of 24hrs, however upon integration of PGA-NPs loaded with VEGF in the hydrogel, sustained discharge in vitro for up to 35 days was observed thereby accentuating its potential use for numerous cardiac applications [59].

Immobilization of placenta derived MSC-EVs onto synthetic scaffolds was also found to promote revascularization [42]. The EVs were immobilized on to the scaffolds using integrin ligand LLP2A, which enhanced attachment of EVs to the scaffold surface. The ECM-EV complexes were found to inhibit endothelial cell apoptosis as well as promote angiogenesis at the ischemic site [42]. Another study, where natural silk-fibroin hydrogels were loaded with stem cell-derived EVs enriched with miR-675 displayed a significant increase in blood perfusion over a period of 28 days when compared to the miR-675 EVs alone in an aging-induced vascular dysfunction murine model [91].

Being a major contributor of deaths worldwide, novel therapeutic options for cardiovascular defects have been extensively researched as these result in excessive heart muscle damage with minimal regenerative potential. The current therapies are mostly mechanical devices that assist the heart functioning however they do not aid in restoring and repairing the damaged heart tissues [92]. Polygycerol sebacate acrylate-based polymers combined with EVs were found to support active release of EVs over a period of 14 days in an in-vivo model of MI. This highlighted the potential of the hydrogel to protect the EVs from being lost during circulation. It was also observed that the polymer remained at the implanted site of the epicardium for over a month without eliciting a significant immune response thereby indicating its potential use for targeted local delivery of therapeutic molecules [93]. Another study compared the efficacy of small EVs administered through a sodium alginate hydrogel to EVs that were systemically injected into an MI murine model [94]. The sEVs incorporated in the hydrogel were found to have sustained release over a period of 14 days that resulted in enhanced angiogenesis at the infarcted site. When compared to the systemically administered sEVs the hydrogel group also drastically abated cardiac apoptosis and fibrosis having an eventual impact on restoring cardiac function [94].

### 4.4. Neuronal Regeneration

Nerve injuries are clinically challenging due to their limited regenerative potential. The conventional treatments mainly included autologous nerve grafts that are now being replaced by artificial nerve guidance conduits (NGCs) due to reduced availability of nerve graft, multiple surgeries for isolating donor graft and risk of developing neuromas. A recent study for the treatment of peripheral nerve injury focused on targeted therapeutics by using alginate hydrogels consisting of laminin- coated Poly(l-lactide-co-glycolide) (PLGA) conduit containing a combination of gold NPs, brain derived growth factor (BDNF) and adipose derived stem cells [69]. The complex system of the hydrogel, NPs, growth-factors and cells were found to significantly promote axonal regeneration and remyelination. They elicited a synergistic effect in a rat sciatic transection model [69]. Another study focused on the delivery of cell adhesive tetrapeptide modified conductive poly(3,4-ethylenedioxythiophene) nanoparticles (PEDOT NPs) from a biocompatible chitin scaffold. The conductive scaffold was found to be highly porous and biocompatible. When introduced in an in-vivo model improved nerve regeneration was observed based on thickness of the regenerated myelin and the area of the muscle fibers. Increased adhesive potential of the schwann cells as well as improved angiogenesis was also observed at the site of injury. Therefore, this electrically active chitin scaffold was suggested to not only be a potential substitute for a nerve guidance conduit but also a beneficial scaffold material for delivery of therapeutics for bone and muscle regeneration [70].

Limited regenerative capacity of spinal cord injuries demands novel therapeutic options. Hydrogels, with their increased water retaining capacity, not only provide the desired mechanical properties and support but are also promising drug delivery vehicles eliciting prolonged therapeutic effects. Mahya et al. [71] demonstrated the consistent delivery of berberine loaded chitosan NPs using a hybrid hydrogel of alginate and chitosan. Combining the gels with endometrial stem cells was also found to elicit a profound effect on the spinal cord regenerative capacity. These complex hydrogel and drug loaded NP systems not only abated the volume of the cavity but also resulted in reduced recruitment of inflammatory cells along with a decline in cell apoptosis and necrosis rates. These results highlighted the sustained release of the berberine loaded NPs resulted in increased neuroprotective effects [71].

### 4.5. Liver and Kidney Regeneration

As stem cell derived EVs are well known to promote healing and regeneration in multiple chronic diseases over time, the sustained release of EVs from hydrogels has been employed in various disease models to analyze the enhanced therapeutic effects of EVs due to increased bioavailability. Mardpour et al. [95], demonstrated the therapeutic effects of embryonic stem cell derived EV (ES-MSC-EVs) laden polyethylene glycol (PEG) macromeres when administered in a chronic liver injury (CLI) murine model. When systemically administered, these EVs were cleared from the system within 24 h; however, the EV loaded PEG macromeres ensured stable release of EVs for 4 weeks in vitro. The prolonged exposure of the fibrotic liver tissue to the hydrogel encapsulated EVs resulted in a drastic decline in fibrosis and apoptosis rates when compared to the systemically administered EVs [95]. MSC-EVs have also been found to alleviate renal injuries, although their application for kidney repair has been challenging due to rapid clearance from the body. However, sustained release of EVs enriched with miRNAs from RGD-Biotin hydrogel in an in-vivo model was found to not only enhance EV retention in the kidneys but also improved the antifibrotic and antiapoptotic effects at functional as well as molecular levels over a period of 7 days [60]. EVs encased in collagen hydrogels were also found to elicit similar effects in an acute kidney injury murine model by not only promoting proliferation and anti-apoptotic effects but also augmenting revascularization at the fibrotic site [96] (Table 2).

## 5. Sustained Delivery of EVs/NPs for Cancer Therapy

Given the potential to load NPs with a variety of anti-cancer agents, and to modify the surface for tumor-targeted delivery, there is immense potential to employ NPs as therapeutics in the cancer setting. Many conventional systemic cancer therapies offer poor targeting and extensive side effects, which could be overcome with the use of NPs [99]. Polymeric conjugates and liposomes are FDA approved nanocarriers for targeted therapy. Although these NPs have huge potential in the cancer setting only few have made it to the market as there are considerable challenges in the release and uptake of these NPs, primarily the issue of accumulation in the liver and spleen when systemically administered in patients. Another limitation is the reduced retention of these particles in the tumor resulting in reduction in its efficacy [99].

Nature’s own nanocarriers, EVs, have also been highlighted as potent carriers of anti-cancer drugs or genetic material based on the unique characteristics of improving drug bioavailability, tumor-targeted delivery, reduced toxicity and protection of therapeutic genetic material/drugs by the lipid bilayer [100,101]. Although EV based therapeutics bear immense potential in the cancer setting, their clinical potential remains limited as a sustained delivery system of EVs is yet to be established [101]. While the number of studies employing hydrogels in the cancer setting is currently limited, this holds immense promise for tumor-targeted delivery of drugs/cargo, potentially enhancing drug availability and minimizing side effects of chemotherapeutic agents [60].

Thermosensitive hydrogels composed of hydroxypropyl cellulose, silk fibroin and glycerol loaded with gambogic acid carrying NPs were found to elicit enhanced therapeutic effects in a murine model of gastric cancer when compared to systemic delivery of the drugs [60]. The constant sustained release of the drug-loaded NPs at the site of the tumor resulted in increased ability to penetrate the gastric tumors and retain therapeutic drug dosages at the tumor site [60]. The increasing demand for controlled drug delivery systems has been mainly due to the limited bioavailability of only a few hours of drug loaded NPs when administered intravenously. The administration of a common chemotherapeutic drug, doxorubicin, when loaded on to micelles through a polymeric hydrogel was found to elicit enhanced anti-cancer effects for a period of 1 week when administered to breast cancer cells in vitro [102]. When treated with low concentrations of doxorubicin NPs a significant decline in the viability of the cancer cells was still noticed thereby highlighting the prolonged bioactivity of the hydrogel-released drug loaded micelles even at low concentrations [61]. Another study assessing the efficacy of siRNA loaded oligopeptide-modified poly(β-aminoester) NPs from polyamidoamine (PAMAM) dendrimer scaffold was found to elicit promising therapeutic effects in a breast cancer murine model [103]. Doxorubicin loaded polydopamine NPs were also shown to elicit tumor ameliorating effects when administered in a murine model over a period of 15days using a self-healing hydrogel [104]. The nanocomposite hydrogel being thermo-responsive was found to be very effective and easy to administer as the gel could be injected in a liquid sate via an intratumoral injection and, when exposed to the NIR laser, the hydrogel heated up, resulting in the release of the drug loaded NPs [104]. Hydrogels and NP/EV complexes could therefore be the way forward for enabling the targeted and sustained delivery of anti-cancer therapeutics.

## 6. Conclusions

Regardless of the disease setting, targeted therapeutics with increased bioavailability and persistence, and reduced toxicity to healthy tissue are a primary requirement. Natural and synthetic EVs and NPs have been explored as a therapeutic option for tissue regeneration and cancer, but clinical application has remained limited due to the lack of sustained delivery systems to ensure localized delivery of efficacious drug doses. Herein, we highlight the prospect of the sustained and prolonged delivery of these nano-sized therapeutic entities from hydrogels for broad applications spanning tissue regeneration and cancer treatment [10,20]. While synthetic NPs can be reproducibly manufactured, the clinical use of EVs was previously limited due to issues associated with the isolation and storage of large quantities of pure and consistent EVs. However, advances in technologies and guidelines that support reproducible EV production, isolation and characterization on a large scale have begun to ameliorate that challenge [17]. The use of bioreactors supports mass EV production through enhanced sheer stress or compression, or exposure to hypoxic conditions to increase EV release [105]. Encapsulation of EVs/NPs in biocompatible hydrogels will support more targeted, tunable delivery and reduce systemic loss of the therapeutic payload, with examples including the controlled release of EVs from scaffolds in response to alterations in pH [89,106].

The release kinetics of nano-sized particles is not only based on the characteristics of the NPs but also the mechanical properties, breakdown rate and stress distribution of the biomaterial. Therefore, optimization of these biomaterials in parallel with the NPs is critical [106]. An in vitro study assessing the controlled release of NPs from hydrogels highlighted the role of electric charge interactions in enabling sustained release of the particles. The superficial charges of NPs and hydrogel mean mesh size were exploited to control NP release [51]. As most of the studies involving the delivery of EVs that use hydrogels are still in the early stages, prolonged release rates are yet to be achieved. This will be a critical milestone in the clinical translation of these hydrogel-EV composites [20]. Although limited studies have been performed to date in the cancer setting, the hydrogel system could potentially support continual NP release for prolonged periods to target metastatic sites and reduce disease progression and/or recurrence. The use of biocompatible hydrogels combined with EVs derived from a compatible cell source (e.g., MSCs) will also limit any immunogenic response. In summary, hydrogels loaded with therapeutic NPs/EVs hold immense promise as cell-free sustained and targeted delivery systems in a range of disease settings. Further understanding of the parameters controlling the release rate of these particles and the efficient transfer of cargo to target cells will be fundamental to success.

## Figures and Tables

**Table 1:** Nanoparticles (NPs) delivered via biomaterial scaffolds for tissue repair in representative examples in various injury models.

Disease	Study Done	NP Details	Drug and Dosage	ROA	BA	Hydrogel Details	Hydrogel Loading	Outcomes	References
Ischaemic Myocardium	In-vitro	PGA-VEGF NPs	VEGF; L-PGA-VEGF; star-PGA-VEGF; (30:1; 50:1)	Hydrogel encapsulated NPs	N/A	HA-TA	Gelation using crosslinkers	↑ Cardiac applications	[59]
Gastric Cancer	In-vivo	Gambogic Acid (GA-NPs)	iRGD (1 mg/mL); GA (0.4 mg/mL)	SC injection	N/A	HPC/SF/Gly	Thermosensitive gelation	↑ Anti-tumor effects	[60]
Breast Cancer	In-vitro	DOX loaded micelles	DOX; 0.7 & 0.14 mg/mL	Cytotoxicity assay	N/A	P(BnN3)-PEG-P(BnN3) and P(DBCO)-PEG-P(DBCO)	SPAAC	↑ Anti-tumor effects	[61]
Wound Healing; Antitumor effects	In-vitro; in-vivo	PEG functionalized CuS NPs	<1 μmol NPs/mg hydrogel	SC injection	14-days	CuS NC	Mixing and Gelation	↑ Tumor inhibition, bacterial eradication, and wound healing	[62]
Thermochemotherapy	In-vitro; in-vivo	PD NPs	DOX; 1:1, *w*/*w* %	IT injection 50 μL of hydrogel	15-days	SP(DMAEMA-co-HEMA-AA)/PEI/PDA NPs	Mixing and Gelation	↑ Drug utilization	[63]
Wound Healing	In-vitro; in-vivo	Silver NPs	1 mM, 2 mM, 4 mM	DA	N/A	Alginate/Gelatin	Mixing and Gelation	↑ wound healing and antimicrobial capacity	[64]
Wound Healing	In-vitro	Silver NPs	110 µg/mL	N/A	N/A	methacrylic acid	Swelling in AGNP solution	↑ Wound healing	[65]
Wound Healing	In-vitro; in-vivo	ZnO NPs; HP-nZnO NPs	0.5%,1%,2% of nZnO & HP-nZnO	DA	N/A	PVA/CS	freeze-Thaw (F-T) method	↑ Re-epithelialization and collagen formation	[66]
Bone Regeneration	In-vitro; in-vivo	CS/CMCS/SDF-1α NPs	5.75 μg of NPs (500 ng SDF-1α)	calvarial defects	N/A	CS/GP	Mixing and Gelation	↑ Bone formation in rat cranial defects	[67]
Articular Cartilage Repair	In-vitro; in-vivo	Chitosan NPs	20% TGF-β1@CS/BMP-2@SF	N/A	N/A	Silk Fibroin	ultrasound crosslinking	↑ chondrogenesis of BMSCs	[68]
Neuronal Regeneration	in-vivo	Chitosan NPs	Au NPs; BDNF; rADSCs	Sciatic gap	N/A	PLGA, Alginate	Electrospinning (PLGA), Alginate (Gelation)	↑ Axonal regeneration and remyelination	[69]
Peripheral Nerve Repair	In-vitro and in-vivo	PEDOT NPs	70, 140, and 210 mg	Sciatic gap	N/A	Chitin	Freeze-Thaw (F-T) method	↑ Sciatic nerve regeneration, ↑ schwann cell adhesion and angiogenesis	[70]
Spinal Cord Repair	In-vitro; in-vivo	Chitosan NPs	Berberine	Lesion sites	N/A	Alginate/Chitosan	Mixing and Gelation	↑ Regeneration of nerve fibers and ↓ vacuolization spaces in the regeneration of SCI	[71]

Abbreviations: ROA—Route of Administration; BA—Bioavailability; DA—Direct Application; N/A—Not Applicable; NPs—Nanoparticles; L-PGA—Linear Polyglutamic Acid; VEGF—Vascular Endothelial Growth Factor; HA-TA—Tyramine-modified Hyaluronic acid hydrogel; SC—Subcutaneous; IT—Intra-tumoral; DOX—doxorubicin; HPC/SF/Gly—Hydroxypropyl Cellulose-Silk Fibroin—Glycerol Hydrogel; SPAAC—strain-promoted azide−alkyne cycloaddition; PEG—Polyethylene Glycol; CuS NC—Copper Sulphate Nano Complex; PD—Polydopamine; SP(DMAEMA-co-HEMA-AA)/PEI/PDA-Four-Armed Star-Shaped Poly(DMAEMA—co-HEMA) [SP(DMAEMA-co-HEMA)]—poly(2-(dimethylamino)—ethyl methacrylate) (PDMAEMA); HP-nZnO—Heparinized nano Zinc Oxide; CS/CMCS/SDF-1α—chitosan/carboxymeymethy-chitosan/SDF-1α; CS/GP-chitosan/β-glycerol phosphate disodium salt (CS/GP); PVA/CS—Poly Vinyl Alcohol/Chitosan; TGF-β1@CS—Transforming growth factor β1 loaded chitosan NPs; BMP-2@SF—Bone morphogenetic protein-2 loaded Silk fibroin Hydrogel; BMSCs—Bone Mesenchymal Stromal cells; Au NPs—Gold Nanoparticles; BDNF—brain—derived neurotrophic factor; rADSCs—Rat adipose–derived stem cells; PEDOT—Poly(3,4-ethylenedioxythiophene; PLGA—Poly(lactic-co-glycolic acid).

**Table 2:** Extra cellular vehicles (EVs) delivered via biomaterial scaffolds for tissue repair in representative examples in various injury models.

Disease	Study Done	EV Details	Cargo and Dosage	ROA	BA	Hydrogel Details	Hydrogel Loading	Outcomes	References
Bone Defects	In-vitro; in-vivo	HGMSCs; PEI EVs	N/A	DA	6 wks	Poly Lactide	Seeded on the hydrogel	↑ osteogenic commitment; bone healing	[41]
Skeletal Regeneration	In-vivo	hPDLSCs-EVs; PEI EVs	N/A	trichotomy	6 wks	Collagen membrane [Evo]	seeded on the collagen membrane	↑ mineralization process, ↑ vascular network formation; osseointegration	[88]
Wound Healing	In-vitro	Royal jelly	2.5 × 10^9^/mL	N/A	7 days	Type I Collagen	Gelation	↑ Wound closure; antibacterial properties	[76]
Chronic Liver Failure	In-vivo	Human ES-MSCs	350 μg	IP	1 month	clickable PEG	Gelation	Enhanced antifibrotic effects vs. conventional bolus injection	[95]
Cartilage Defects	In-vivo	hUC-MSCs	miR-23a-3p; 10 × 10^8^ particles/mL	DA	N/A	Gelma/nano- clay	Gelation	↑ cartilage regeneration by activating PTEN/AKT signalling pathway	[90]
Acute Kidney injury	In-vivo	hP-MSCs	miRNA let-7a- 5p; 100 μg/mL	IR	7 days	RGD-biotin (biotin-GFFYGRGD)	Gelation	↑ renal function; proliferation, antifibrosis, antiapoptosis; proautophagy ↓ tubular injury	[97]
Acute Kidney injury	In-vivo	hPMSC	100 μg in 100 μL of gel	IR	6 days	Collagen Matrix	Gelation	↑ proliferation, anti-apoptosis, and angiogenesis	[96]
Vascularization	In-vitro	PMSC	5 × 10^6^ EVs	seeded on scaffolds	N/A	Electrospun scaffolds (PLLA+PCL)	Immobilization of LLP2A	↑ EC angiogenesis and prevented EC apoptosis	[42]
Diabetic skin wounds	In-vivo	hGMSC	150 µ g EVs/wound	DA	2 wks	chitosan/silk	Injected into the hydrogel	↑ re-epithelialization, deposition and remodelling of ECM, angiogenesis and neuronal ingrowth	[40]
Skin wounds	In-vitro; in-vivo	hUCMSC	miR-21, miR-23a, miR-125b; miR-145; 100 µg EVs	DA	N/A	Hydro Matrix	Gelation	↑ anti-scarring effect around each wound prevents α-SMA expression and scar formation.	[81]
Articular Cartilage Lesions	In-vitro; in-vivo	hiPSC-MSC	1 × 10^11^/mL	IA	N/A	PIC	Gelation	↑ Articular cartilage regeneration	[35]
Wound Healing	In-vivo	ADSCs	22 µg	IP	N/A	Alginate	Gelation	↑ wound closure, reepithelization, collagen deposition, angiogenesis	[82]
Vascular dysfunction	In-vivo	UMSCs	miR-675 11 mg/mL	Femoral artery	N/A	Silk fibroin	Sonication; Gelation	↑ blood perfusion, stability & retention of EVs	[91]
Bone regeneration	In-vitro; In-vivo	hucMSC	50 μg/μL	DA	N/A	CHA/SF/GCS/DF-PEG	Gelation	↑ bone healing; BMP2 deposition, bone collagen deposition; maturation and angiogenesis	[89]
Endometrial Regeneration	In-vivo	UCMSCs	3 × 10^11^ EVs/mL	DA	15 days	Collagen	dropwise on the scaffold for infiltration	↑ endometrium regeneration, collagen re- modelling, expressions of Erα & PR in the regenerated endometrium	[98]
Cardiac Repair	In-vitro; In-vivo	iPSC-Pg	Trehalose; 4.5 × 10^10^ EVs/mL	implanted on the polymers	N/A	PSA	Light activated cross linking	↓ degradation of EVs from without inducing an inflammatory response	[93]
Myocardial Infarction	In-vivo	MSCs	80 μg of EVs	IM	14 days	Sodium Alginate	Gelation	↑ angiogenesis and scar thickness ↓ cardiac apoptosis and fibrosis	[94]

Abbreviations: ROA—Route of Administration; BA—Bioavailability; DA—Direct Application; N/A—Not Applicable; Wks—weeks; HGMSCs—human gingival Mesenchymal Stromal Cells; hPMSCs—human placenta—derived MSCs; IR—Intra Renal; PLLA—PolyLactic Acid; PCL—Polycaprolactone; EC—Endothelial Cell; α-SMA—α-smooth muscle actin; hiPSC-MSC—human induced Pluripotent stem cell derived MSCs; IA—Intra articular; PIC—photoinduced imine crosslinking; ADSCs—Adipose derived stromal cells; iPSC-Pg—Human induced pluripotent cardiac progenitors; PSA—Polyglycerol sebacate acrylate; PEI—Polyethyleneimine; hPDLSCs—Human Periodontal-ligament stem cells; Evo—Evolution; ES-MSCs—Embryonic Stem cell derived MSCs; hUC-MSCs—Human Umbilical Cord derived Mesenchymal stromal cells; IP—Intra Peritoneal; PEG—polyethylene Glycol.

## Data Availability

Not applicable.
